# Endovascular Preparation With Innovative Custom-Made Stent-Graft Before Kidney Transplantation: The Solution for Patients With Hostile Iliac Calcification

**DOI:** 10.3389/ti.2024.13486

**Published:** 2025-01-08

**Authors:** Matthieu Arsicot, Raffaele Pio Ammollo, Marine Bordet, Leila Dehina-Khenniche, Olivier Thaunat, Nellie Della Schiava, Antoine Millon, Emilien Seizilles De Mazancourt, Lionel Badet, Xavier Matillon

**Affiliations:** ^1^ Service de Chirurgie Vasculaire et Endovasculaire, Hospices Civils de Lyon, Lyon, France; ^2^ Centre de Référence des Infections Vasculaire Complexes (CRIVasc Network), Hospices Civils de Lyon, Lyon, France; ^3^ Laboratoire de Genie Electrique et Ferroélectrique (LGEF), Institut National des Sciences Applicquées (INSA)-Lyon, University Lyon, Villeurbanne, France; ^4^ Service de Transplantation, Néphrologie et Immunologie Clinique, Hôpital Edouard Herriot, Hospices Civils de Lyon, Lyon, France; ^5^ Service d’Urologie, Hôpital Saint Louis, Paris, France; ^6^ Service d’Urologie et de Transplantation, Hospices Civils de Lyon, Lyon, France

**Keywords:** atherosclerosis, graft survival, kidney transplantation, vascular calcification, vascular surgical procedures, iliac endoprothesis, endovascular procedures

## Abstract

The increasing age of patients with end-stage renal disease raises the issue of hostile arterial access for transplantation, with technical difficulties associated with clamping and suturing the iliac artery. Some of these patients - who theoretically represent those who would benefit the most from transplantation in terms of mortality - are contraindicated because of anatomical and medical issues. In this context, a specific endovascular device called EndoPreKiT (Endovascular Preparation for Kidney Transplantation) has been designed, enabling arterial access for transplantation via a mini-invasive procedure. It consists of a woven Dacron supported by self-expanding nitinol rings, ensuring anchorage and allowing arterial clamping. The middle part of the anterior face of the device is stentless, enabling the anastomosis directly onto the Dacron once the calcified artery wall has been removed. After a cadaveric study validating its technical feasibility, such device was successfully implanted in 10 patients considered unfit for transplantation due to severe wall calcification. Two of them have been successfully transplanted with excellent outcomes after 13 and 3 months of follow-up. EndoPreKiT device may be a significant breakthrough in transplant surgery, that could expand the horizon of eligibility to include even the most fragile patients with challenging arterial access.

## Graphical Abstract

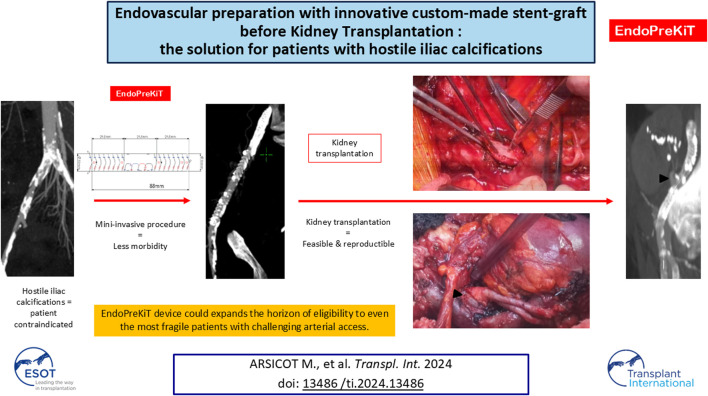

## Introduction

The increasing age of patients with end-stage renal disease (ESRD) raises the issue of hostile arterial access for transplantation, since the perturbations of phosphocalcic metabolism associated with chronic renal disease are a key factor favouring mediacalcosis [1]. Because of the technical difficulties involved in clamping and suturing the iliac artery, many patients are contraindicated for Kidney Transplantation (KT) [2, 3].

In such cases, an aorto-iliac bypass may be offered, providing a space for clamping and anastomosis at the time of subsequent KT. However, the morbi-mortality associated with this procedure is substantial (estimated 10%–30% [4]) in these high-risk cardiovascular patients [5, 6]. Moreover, KT is usually made more difficult in case of previous abdominal surgery. However, these high-risk cardiovascular patients who are considered unfit for transplantation, are precisely those who would benefit most from such intervention in terms of survival [7]. In this context, a specific endovascular device has been designed to prepare the arterial access for kidney transplantation using a minimally invasive endovascular procedure.

This paper aims to delineate the development of this innovative device, from its conceptualization to rigorous validation via cadaveric studies. Furthermore, we offer insights garnered from our initial clinical endeavours, encompassing the implantation of the device in ten patients previously deemed unsuitable for transplantation, and the inaugural kidney transplantation in utilising the device in two of them.

## Materials and Methods

### EndoPreKiT Device

**FIGURE 1:**
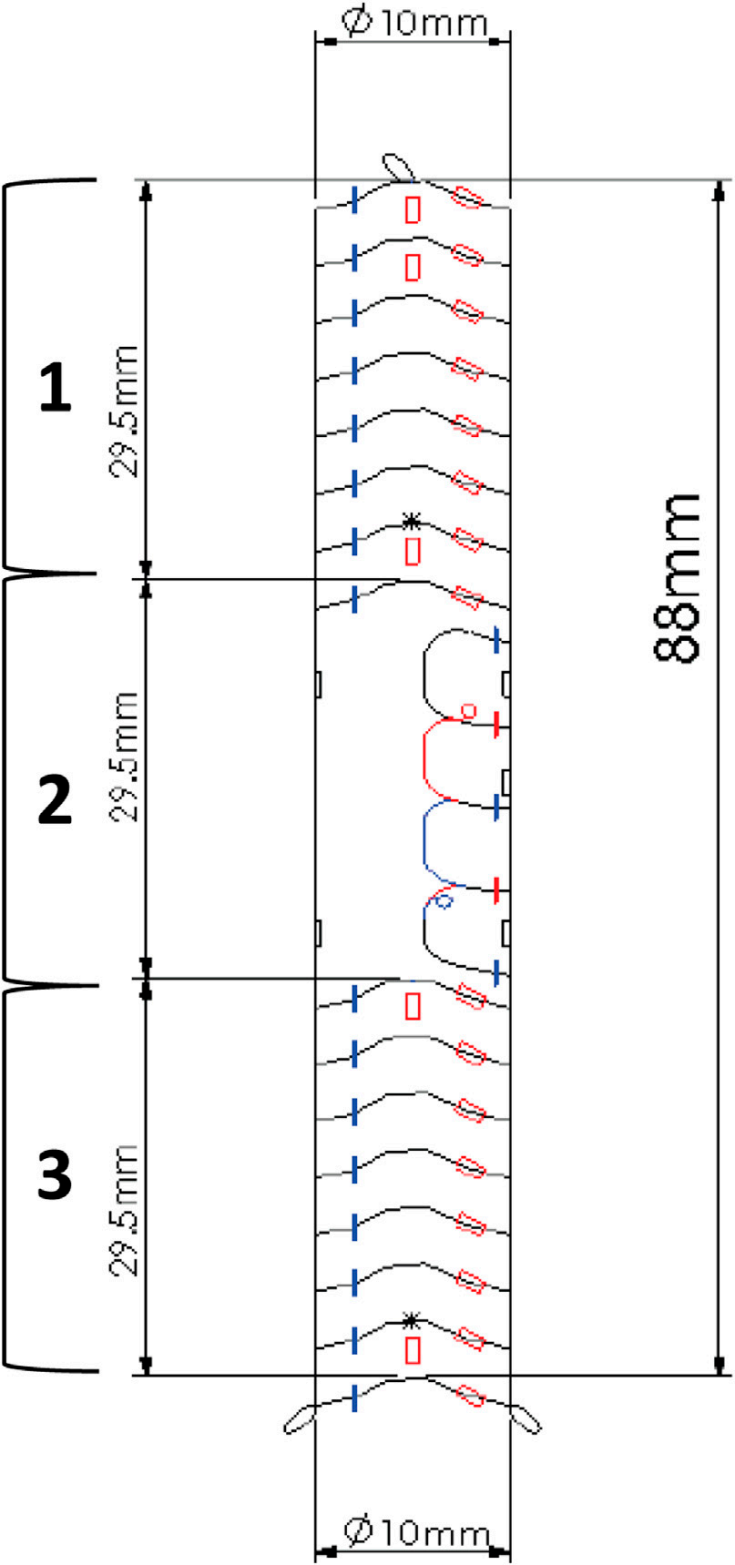
Example of an EndoPreKiT planning design, left side view. The proximal and distal parts (**1** and **3**) are reinforced by the stent rings for sealing and clamping. The anterior face of the middle part (**2**) is free of any stent to receive the future renal artery anastomosis.

The Anaconda endoprosthesis, produced by Terumo Aortic is a custom-made device (Figure 1). Its use is currently approved for use in aortic and iliac artery aneurysmal disease. Under the regulations of tailor-made Medical Devices, EndoPreKiT is a custom made device produced by Terumo Aortic at the surgeon’s request on the basis of the patient’s specific anatomical criteria and for a single and precise indication, that is the vessel preparation for kidney transplantation in case of severe iliac atherosclerotic occlusive disease. It consists of a woven Dacron supported by self-expanding nitinol rings, which seal the device proximally and distally (Figures 1.1, 1.3) to have sealing and clamping. In the EndoPreKiT module, the middle segment (Figure 1.2) is lined with half stents in the posterior wall only to avoid the collapse and thrombosis of the graft; the stent-less anterior wall allows graft anastomosis directly on the Dacron, after removal of the calcified artery wall.

The manufacturer allows the combination of different proximal and distal diameters, with a minimum total length of 88 mm. The endoprosthesis is designed for deployment in the external iliac artery without internal iliac artery coverage, but it can be placed more proximally depending on the patient’s anatomy. Several radiopaque markers were placed on the device to ensure deployment in the correct orientation, in which the platform receiving the graft anastomosis is anterior (Figure 1). These markers will also allow fluoroscopic detection of the anastomosis site during KT.

### Proof of Concept: Cadaveric Study

A preliminary study on human cadaver was carried out to verify the technical feasibility of endovascular implantation as well as the suturability of the device, in an anatomical dissection laboratory equipped with a C-arm on a human female cadaver of 83 years of age. The body was not perfused, as we did not aim to test device patency. This study was financed by internal funding.

### EndoPreKiT Implantations in Patients on the Waiting List

**FIGURE 2:**
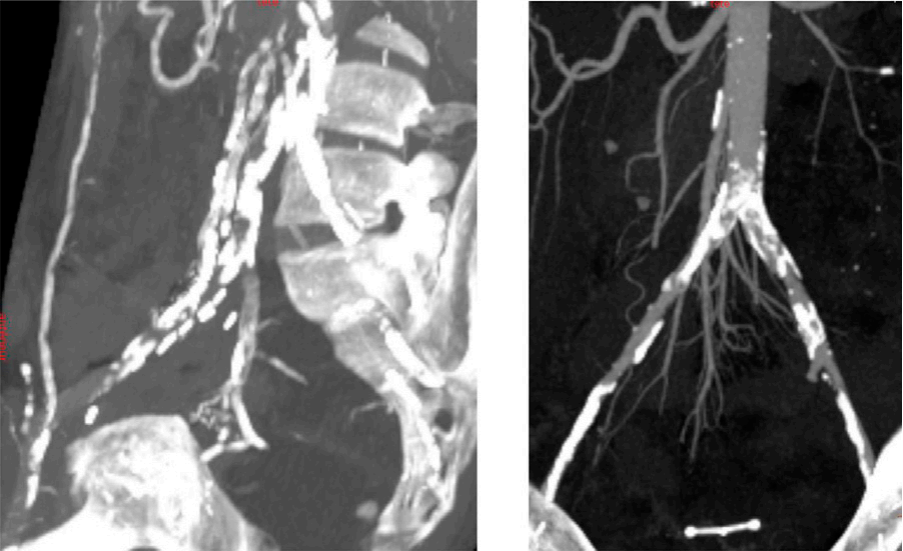
Two examples of massive aorto-iliac calcification with technical contraindications to kidney transplantation.

After multidisciplinary team discussions, patients can be judged unfit for KT and are contraindicated due to massive aorto-iliac calcifications (Figure 2). A total of ten patients (median age: 64 years old, range 43–74; 50% male) were contraindicated and deemed suitable for EndoPreKit implantation, from January 2021 to January 2024, due to arterial access problems: calcifications (impossibility to clamp and suture the future kidney graft), with or without stenosis. If significant stenoses were associated, additional stenting during the procedure was planned to ensure flow through the endoprosthesis. In this case, the custom-made endoprosthesis could be adapted to position the graft platform before or after the stenosis. The institutional ethics committee allowed EndoPreKiT implantation as the procedure in this extremely selected population outside of a phase 1 study because in the context of a therapeutic impasse. Informed consent was obtained from all ten patients undergoing the procedure over a period of 34 months (April 2021 – February 2024). A multicenter study is ongoing (NCT06677437). As authorized by French law, this is a retro and prospective data study. This study was financed by internal funding. Clinical characteristics are described in Table 1. This study was validated by the methodological and regulatory support teams of the HCL sponsor, as well as by the Scientific and Ethics Committee of the Hospices Civils de Lyon (autorisation no. AGORA 23-5431 and avis CFE 23-431).

**TABLE 1:** Clinical characteristics of patients treated with EndoPreKiT module.

Patient number	Sex	Age at EndoPreKiT implantation	HTA	Diabetes	Dyslipidemia	Obesity	Respiratory failure/COPD	CAD	ASA score	Preoperative anticoagulant/antiplatelet therapy	Dialysis type	Type of renal insufficiency	Previous renal transplantations	Number of previous renal transplantations	Previous iliac interventions	Previous abdominal interventions	Preoperative GFR
1	F	43	-	-	-	-	-	✔	3	DAPT	HD	Nephrotic syndrome	No	-	✔	✔	19
2	F	54	✔	-	✔	✔	-	-	3	SAPT	HD/PD	Interstitional nephropathy from uretral reflux	✔	1	-	-	7
3	M	64	✔	✔	✔	-	-	-	3	SAPT	HD	Diabetic nephropathy	No	-	✔	-	6
4	M	72	-	-	✔	-	-	✔	2	AVK	HD	Primary glomerulonephritis	✔	2	-	✔	14
5	M	66	✔	✔	-	-	-	-	3	SAPT	HD	Nephroangiosclerosis/iatrogenic nephropathy	No	-	-	✔	13
6	F	70	✔	✔	✔	✔	-	-	3	SAPT	HD	Diabetic nephropathy	No	-	-	✔	11
7	M	74	✔	-	✔	-	-	✔	3	SAPT + AVK	HD/PD	Interstitial nephropathy	No	-	✔	✔	4
8	M	64	✔	-	-	-	✔	✔	3	SAPT	HD	Iatrogenic nephropathy	No	-	-	-	13
9	F	46	✔	-	-	-	-	-	2	SAPT	HD	ADPKD	No	-	-	-	9
10	F	62	✔		-	-	-	✔	3	SAPT	HD	Primary glomerulonephritis	✔	1	✔	✔	5
Total/Mean		61,5	8	3	5	2	1	5	-	-	-	-	3	-	4	7	-

Abbreviations: HTA, arterial hypertension; COPD, chronic occlusive pulmonary disease; CAD, coronary artery disease; eGFR, estimated glomerular filtration rate; DAPT, double antiplatelet therapy; SAPT, single antiplatelet therapy; AVK, anti-vitamin K; HD, hemodialysis; PD, peritoneal dialysis.

### First Two Cases Patients

The first transplantation was performed on a 70-year-old woman, with a Nephroangiosclerosis (NAS) and diabetic nephropathy, on haemodialysis since 2010. She was considered unsuitable for KT because of circumferential aorto-iliac calcifications and a hostile abdomen due to previous iterative surgeries, with haemorrhagic and infectious complications after a gastric bypass for class III obesity.

The second transplantation was performed on a 37-year-old woman with an autosomal dominant polycystic kidney disease. She was treated with peritoneal dialysis since April 2021. She was also considered unsuitable for KT because of bilateral circumferential aorto-iliac calcifications.

## Results

### Cadaveric Study

**FIGURE 3:**
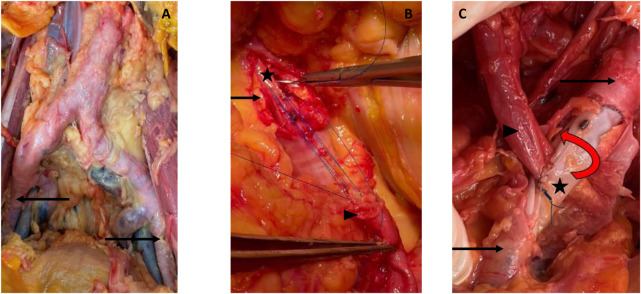
Cadaveric study. **(A)** Calcified cadaveric iliac arteries (black arrows). **(B)** Anastomosis of the renal artery (black arrowhead) to the anterior middle segment of the endoprothesis (black star) after removal of the anterior wall of the iliac artery. Parachute tension suture to test the resistance of the graft. **(C)** Final control of the end-to-side anastomosis with a twist to test the resistance of the graft.

We used two custom-made endovascular legs dedicated to kidney transplantation (Anaconda^®^ Vascutek, Terumo Aortic), of 10 mm of both proximal and distal diameter, and 100 mm of length (30, 30, and 40 mm proximal, medium, and distal parts, respectively) Figure 3.

### The First Step Vessel Exposure

Through a conventional laparotomy, iliac vessels were dissected on both sides, showing a high degree of calcification. After an open bilateral exposure of the femoral arteries, the two superficial femoral arteries (SFA) were retrogradely punctured with a 16G needle. Common femoral artery puncture was avoided due to major circumferential calcifications. Under fluoroscopy, the aorta was catheterised with a Terumo®O35 260 cm stiff guide wire. Through a 65 cm multi-graded straight catheter, a straight O35 180 cm ultra-stiff guidewire (Lunderquist, Cook Medical) was positioned in the descending thoracic aorta. We used a 16Fr sheath (Dryseal, Gore Vascular) to verify the navigability in the distal external iliac artery (EIA). As no friction was encountered, no preventive dilatation was necessary. The stent graft was thus placed into the proximal EIA. The internal iliac artery was identified on the angiogram and the correct position of the stent graft was verified according to the alignment of the markers. Once the device was completely released, a post-dilation with a 10 × 40 mm balloon was performed along the entire length of the device for impaction and sealing. The same procedure was repeated on the contralateral iliac side. The final arteriographic control was satisfactory on both sides.

### Second Step: Kidney Autotransplantation on the Endograft

Through the laparotomy, a conventional bilateral nephrectomy was performed. The renal arteries were only slightly calcified.

Using fluoroscopy guidance, the markers of the middle segment were identified, and the vessel was marked with a sterile pencil. Through a longitudinal arteriotomy, the stent-less anterior part of the endograft was exposed. After an external cross-clamping of both artery and graft on the proximal and distal portions of the device, a longitudinal incision of the anterior surface of the middle segment of the endograft was carried out to allow the suturing of the renal artery, with an end-to-side suture (Figure 3). During suturing time, we did not experience any difficulty in needle penetration through the graft; there was no tissue weakness, no tearing, and no need to reinforce the suture line. Anastomosis was then checked with a high-pressure serum injection through the femoral introducer sheath.

### EndoPreKiT Implantations in Patients on the Waiting List: Endovascular Procedure

**FIGURE 4:**
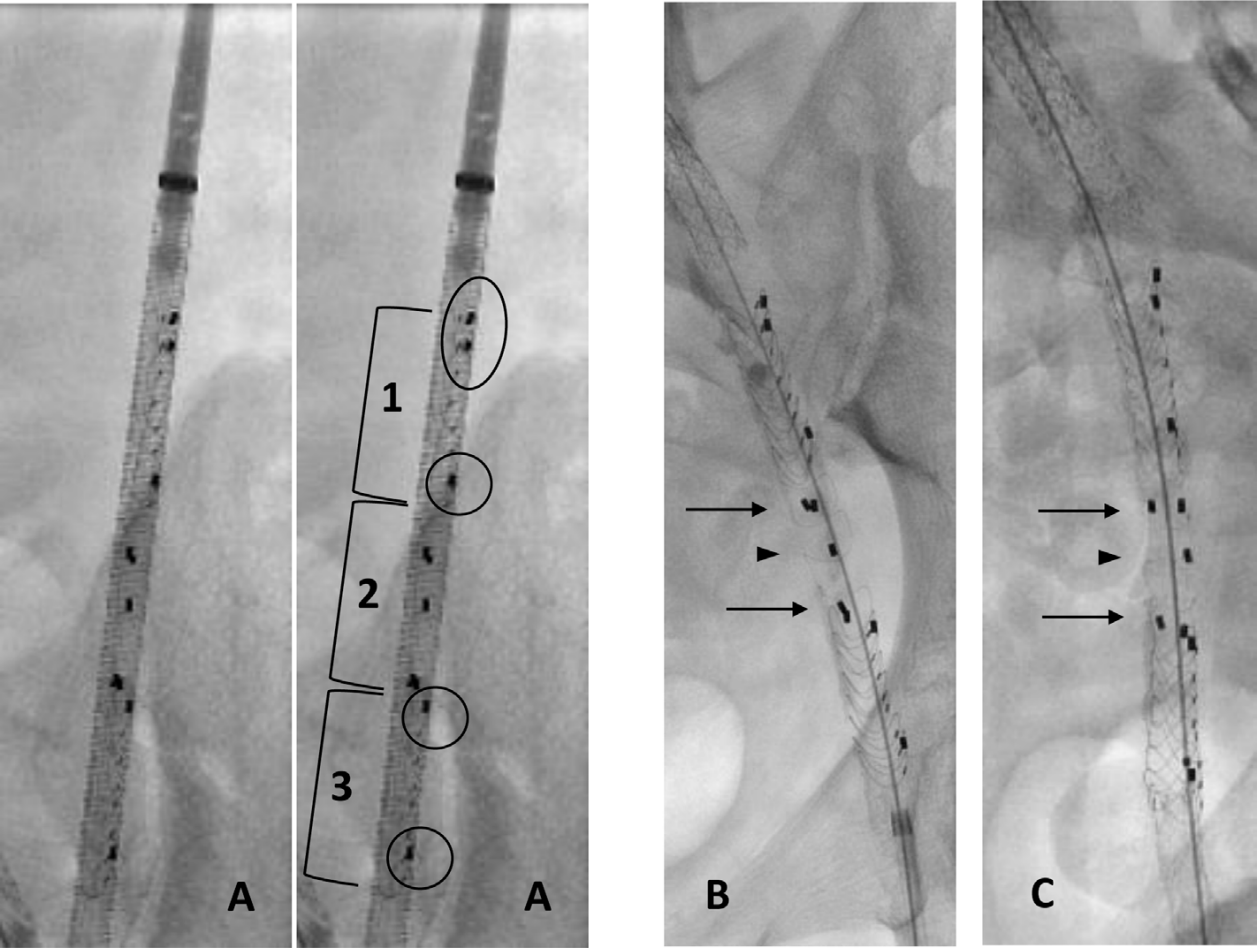
EndoPreKiT during implantation with angiography. **(A)** Front view before deployment: **1**: proximal part of endoprosthesis with two first left markers to avoid loading errors (*black oval*); only one distal lateral marker on the patient’s left (*black circle*). **2**: middle part of the endoprosthesis, waiting for future anastomosis, marked by two pairs of anteroposterior markers. **3**: distal part of endoprosthesis with only one proximal and one distal lateral marker on the patient’s left (*black circle*). **(B)** Front view after deployment: superposition of the two pairs of anteroposterior markers to place the part free of any stent anteriorly (*black arrows*). **(C)** Lateral view after deployment: good opposition of 2 pairs of anteroposterior markers; the single marker in the middle is well posterior (*black arrowhead*).

All the procedures were performed by the same experienced operator in a hybrid room and fusion imaging (Figure 4). Open femoral access was necessary for one patient to perform a prosthetic femoral bypass, under general anesthesia. The remaining 9 cases were performed under local anesthesia, through a percutaneous femoral access (3/9 common; 6/9 superficial) obtained with ultrasound guidance and preclosed with two percutaneous closure devices (Proglide; Abbott Vascular Inc., Santa Clara, CA, United States).

The procedure systematically began with predilatation of the iliac artery using an 8 mm balloon. The stent graft was then advanced into the iliac artery, and the precise alignment of the stent graft was verified thanks to the radio-opaque markers, as follows:

- From the front, five markers positioned on the left side of the stent mark the beginning and end of the proximal and distal stents. A PAIR of proximal marker ensures that the stent graft was loaded correctly into its launcher. At the opposite distal part of the endoprothesis is present only one lateral marker on the patient’s left (Figure 4A).
- Middle part of the endoprosthesis, waiting for future anastomosis, marked by two pairs of anteroposterior markers. From the front the three posterior and two anterior markers of the middle segment must be aligned to ensure correct orientation of the free stent platform that will receive the graft anastomosis (Figure 4B). Figures 4B, C shows front view after deployment (superposition of the two pairs of anteroposterior markers to place the part free of any stent anteriorly) and lateral view after deployment (good opposition of 2 pairs of anteroposterior markers). Note that the single marker in the middle is well posterior.

In the case of the patent internal iliac artery, this was identified by angiography and the stent graft deployed in the external iliac (in 5/10 patients, right). Finally, balloon angioplasty was systematically performed to obtain expansion and correct apposition of the stent graft to the vessel.

Technical success was 100%, with a mean operative time of 98.7 min (range: 52–260), mean radiation dose of 2907.0 μGy/m^2^ (range: 465.5–9722.6), mean radiation time of 15.62 min (range: 9.7–25.2) and a mean 45.8 mL of contrast volume injection (range: 20–71). No device misalignments nor vessel ruptures were seen intraoperatively. No deterioration in renal function was observed in the non-dialyzed patients.

In 7/10 patients, a planned adjunctive procedure was necessary: 1 common femoral artery prosthetic bypass for access creation due to severe atherosclerosis enabling common femoral artery percutaneous access; 1 ilio-femoral stenting to allow device sheath progression (paving/cracking); 4 common iliac stent deployments to improve inflow due to significant stenosis; 1 hypogastric artery embolization and common iliac stenting to create a safe proximal landing zone ensuring correct sealing.

No postoperative transfusion was needed. All patients were discharged without complications after a median hospital stay of 2 days (range: 2–4), under double oral antiplatelet therapy of 75 mg acetylic acid plus 75 mg clopidogrel for 3 months, and single antiplatelet therapy thereafter. All patients underwent CT angiography and US Doppler at 1-month post-procedure to remove the contraindication to KT.

### Kidney Transplantation on EndoPreKiT: The First Two Cases

**FIGURE 5:**
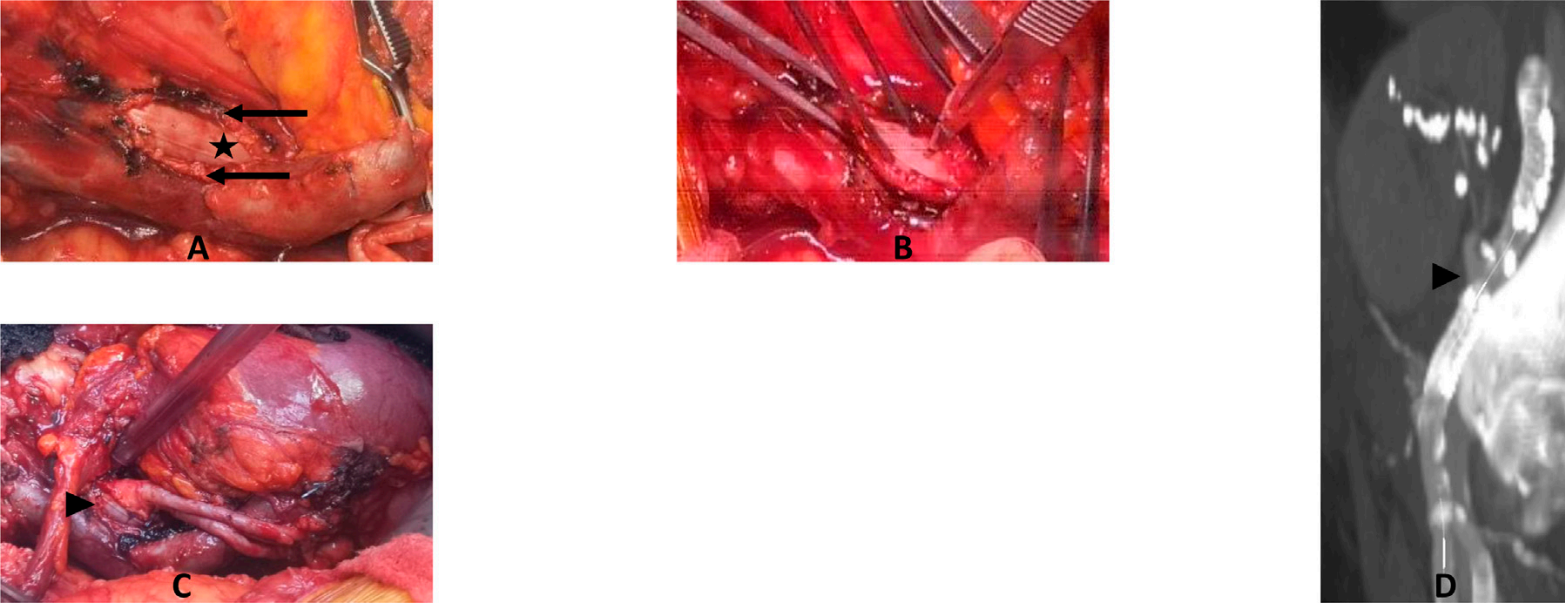
*In vivo* kidney transplantation. **(A)** Arteriotomy and removal of the anterior wall of the iliac artery (*black arrow*) for EndoPreKiT exposition (*black star*) after venous anastomosis. **(B)** Prothetotomy on the anterior part of the middle segment without stent, during a cross-clamping of the iliac artery, ignoring calcifications. **(C)** *In vivo* kidney transplantation. Renal artery anastomosis (*black arrowhead*), venous anastomosis and ureteroneocystostomy done. **(D)** Angiographic CTscan: arterial anastomosis control (*black arrowhead*) with endoprosthesis’s markers.

The first KT was performed 121 days after the implantation of *EndoPreKiT* using a graft with Extended Criteria (ECD) and Donation after Brain Death Donor (DBD) (Figure 5). The right external iliac artery was dissected through a retroperitoneal access. A longitudinal arteriotomy of about 3 cm and the partial ablation of the superior vessel wall allowed the exposure of the middle segment of the device, localised under fluoroscopy. After 100 mg acetylsalicylic acid and 25 UI/kg IV heparin infusion, the iliac artery was cross-clamped at the level of the proximal ring segment. At the distal part, we had to use an endoclamping technique with ballon due to insufficient cross-clamping. Then, arterial anastomosis was performed on the graft with a 6-0 Prolene overlock, with a cold and warm ischemia time of 11.5 h and 37 min, respectively. There were no endoleaks at the end of the procedure, the nitinol rings had no clamping lesions, and no balloon dilation was necessary. Total blood loss was 400 mL and operative time 157 min. After an ICU unit stay of 3 days, spontaneous diuresis was obtained on the 5th day and the double J stent was removed on the 9th day postoperatively. There was no Delayed Graft Function (no need for dialysis during the first week after transplantation). The patient was dismissed under 75 mg PO acetylsalicylic acid after a total hospital stay of 13 days, with a serum creatinine of 175 μmol/L; eGFR 25 mL/min/1,73 m^2^). A non-infected peri-vesical hematoma drainage was necessary 29 days after KT, with a total hospital stay of 15 days.

Follow-up Doppler ultrasounds performed at 1, 2, 3, 4, 7, and 8 months postoperatively, all showed a good intra-parenchymatous vascularisation pattern, as well as a patent graft, without in-stent or anastomotic stenosis. The CT scan performed at the time of the peri-vesical hematoma showed no complications of the EndoPreKiT module or vessel anastomoses. At the last follow-up visit (13 months), graft function was quite stable (creatinine 200 μmol/L; eGFR 20 mL/min/1.73 m^2^).

Our second KT was performed 133 days after the implantation of EndoPreKit. It was an ABO-incompatible living-donor KT. The same surgical technique was performed. EndoPreKiT was cross-clamped: no endoclamping was required. Cold and warm ischemia times were 260 and 51 min, respectively. Total blood loss was 300 mL, and total operative time was 146 min. After a total hospital stay of 7 days, she was dismissed under PO acetylsalicylic acid 75 mg PO, with an excellent graft function (creatinine 70 μmol/L; eGFR >90 mL/min/1.73 m^2^).

Day 0, 1 and 2 months post-operative Doppler ultrasounds showed adequate intra-parenchymatous vascularisation pattern. During the first 2-month follow-up, graft function remained stable without major or minor surgical complications.

## Discussion

This original work confirms the proof of concept of a device, usually used in another situation, which could make some patients eligible for KT in case of severe aorto-iliac calcification.

The number of patients unfit for KT due to severe peripheral artery disease (PAD) is increasing as a consequence of the demographic evolution of patients on the waiting list of KT and of the organ shortage [8]. According to the 2020 Kidney Disease Improving Global Outcomes (KDIGO) Guidelines on the Evaluation and Management of Kidney Transplantation, peripheral artery disease is one of the main areas of examination before such intervention [3]. A recent survey among 939 kidney transplant surgeons has shown a large variability in both diagnosis and treatment of peripheral arterial disease in this setting: 67.7% of respondents rated technical problems as the most important concern, followed by increased mortality risk because of cardiovascular comorbidity and ethical issues [9].

However, KT in older patients and patients with severe aorto-iliac calcification, seems to improve patient and graft survival compared to the renal replacement therapy population [10]. Although it is a recognised source of surgical complexity, severe aorto-iliac artery disease is not an absolute contraindication for kidney transplantation. It is generally considered that at least 3 cm of a disease-free vessel is needed to perform a vascular anastomosis and that in the absence of this criteria, a procedure for vessel preparation (aorto-femoral bypass or iliac endarterectomy) is needed in about 3% of kidney transplant recipients [11, 12].

Particularly in younger patients judged unfit for KT, it is important to offer a solution to overcome severe aorto-iliac calcifications. In preparation for subsequent KT, the aortobifemoral bypass has always been considered the only technique available [10]. In case of the need for vessel preparation, a vascular open surgery can be performed simultaneously or preventively to a kidney transplant [13]. A simultaneous procedure may be beneficial in patients with a high cardiovascular risk, and may as well limit the risk of pretransplantation HLA sensitisation due to blood transfusions [14]. On the other hand, a preventive surgery is associated with a lower risk of kidney thrombosis and vascular prosthesis infection, and benefits from a better stabilisation of the prosthesis within surrounding tissue before transplantation. In the absence of clear guidelines about the optimal delay between a preventive vascular surgery procedure and the transplantation, an interval of less than 1 year is generally considered advisable [13].

In the field of aortoiliac artery disease, endovascular surgery has broadly shown an advantage of reduced morbidity and mortality compared to open surgery, especially in frail patients [15]. In kidney transplant recipients, calcification progression is speeded up by phosphocalcic metabolism changes, and a previous bare metal stent placement makes impossible future arterial sutures.

We designed the EndoPreKit from an original custom-made stent graft whose indication has been changed to make KT possible in this clinically complex population, characterised by a very high cardiovascular risk, through a solution which would appear to be simple, of low-morbidity, reproducible and effective. EndoPreKiT also makes it possible to perform KT under simple and reproducible surgical conditions. To our knowledge, this is the world’s first experience with a custom-made endoprosthesis in the field of transplantation, as it is already known for abdominal aortic aneurysm complex surgery.

The EndoPreKiT module has been conceived to limit most of the technical pitfalls of both open and endovascular arterial preparation of kidney transplantation. The percutaneous device placement leaves the abdomen “free” for any further surgical procedure and can be performed on an outpatient basis, which significantly improves clinical tolerance. Of note, since ESRD patients are already under haemodialysis for the majority, a contrast-enhanced CT scan can be safely performed to proceed to stent-graft sizing. Due to its nitinol stent structure, the EndoPreKiT module is supposed to show the same good long-term patency rates observed for the newer generations of Anaconda iliac limbs [16, 17]. Moreover, as found during our cadaver study, the middle anterior segment of EndoPreKiT shows excellent properties in terms of suture. In our first two cases in living patients, we also confirmed a good sealing of the stent graft to the arterial wall without endoleaks. Cross-clamping of the arterial axis at the proximal and distal part was safely performed without device collapse, ring fracture or residual stenosis requiring balloon angioplasty or re-stenting.

Our study has some limitations. The device in itself can be an obstacle: the 16 Fr sheath progression can be difficult in case of severely calcified arteries, requiring an additional peroperative angioplasty. This device has a cost, its production usually requires 6 weeks, and should be implanted at least 3 months before KT, the duration of the dual antiplatelet therapy to obtain the full endothelialisation of the stent-graft. Transplant surgeons must be aware of the theoretical risk of proximal or distal endoleak, which can be managed with complementary procedures (balloon angioplasty, relining with covered stent…). Other limitations are the study design, its low power and its non-comparative nature. Due to the small sample size, it was not possible to measure the incidence of endoleaks, stent fractures or migration. Further studies are required to assess the long-term patency of the device and other complications.

## Conclusion

The EndoPreKiT device could mark a substantial leap forward in the landscape of kidney transplantation, offering a minimally invasive alternative that extends treatment options to previously marginalised patients with compromised iliac access. Further studies are warranted to comprehensively assess its applicability, long-term efficacy, potential enhancements to quality of life, and overall impact on healthcare expenditure. These endeavours are required to fully comprehend the transformative potential of this innovation and optimise its integration into clinical practice.

## Data Availability

The raw data supporting the conclusions of this article will be made available by the authors, without undue reservation.
